# The Surgical Strategy for Progressive Dilatation of Aortic Root and Aortic Regurgitation After Repaired Tetralogy of Fallot: A Case Report

**DOI:** 10.3389/fcvm.2022.840946

**Published:** 2022-05-03

**Authors:** Shuaipeng Zhang, Haiyuan Liu, Xiangyu Wang, Shaojun Huang, Chengxin Zhang

**Affiliations:** Department of Cardiovascular Surgery, First Affiliated Hospital of Anhui Medical University, Hefei, China

**Keywords:** aortic root, tetralogy of Fallot, left ventricular outflow tract, cardiopulmonary bypass, cardiac surgery

## Abstract

It has been found that postoperative progressive dilatation of aortic root is not rare for adult patients with repaired Tetralogy of Fallot (TOF), which leads to severe aortic regurgitation or even fatal dissection. Therefore, clinically, surgical treatment for both regurgitated aortic valve and dilated root is needed based on preoperative assessments and individual treatment strategies.

## Introduction

Clinically, how to manage and prevent structural abnormalities for both aortic valve and root after repaired TOF (rTOF) has been the predominant point in the surgical field, although, it has been reported that compared with the incidence of postoperative aortic aneurysm, which is the dilatation of aortic root, is relatively lower (12 vs. 9%) (1). Currently, fewer case reports of surgical treatment focusing on how to handle postoperative dilatation of aortic root and aortic regurgitation (AR) after smoothly and reasonably has been shown (2, 3). For our case report, the David I plus repaired aortic valve procedure, combined with simultaneous reconstruction of the left ventricular outflow tract (LVOT), were selected as the surgical option by individual preoperative assessments, and the postoperative outcome of this patient is satisfactory. Hence, it is valuable to share our surgical experience as follows.

## Case Presentation

**Figure 1:**
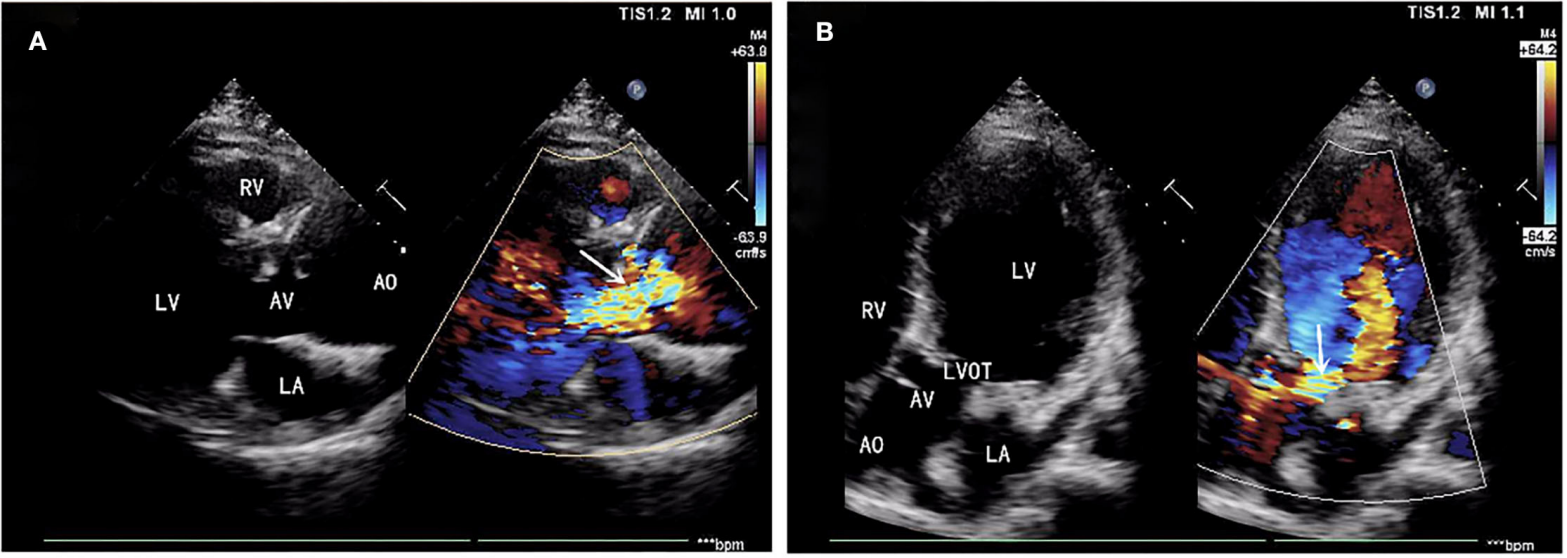
**(A)** Aortic valve insufficiency complicated with severe aortic regurgitation (AR). **(B)** Local obstruction of left ventricular outflow tract (LVOT) (gradients of LVOT 23 mmHg). AR, aortic regurgitation; LVOT, left ventricular outflow tract.

**Figure 2:**
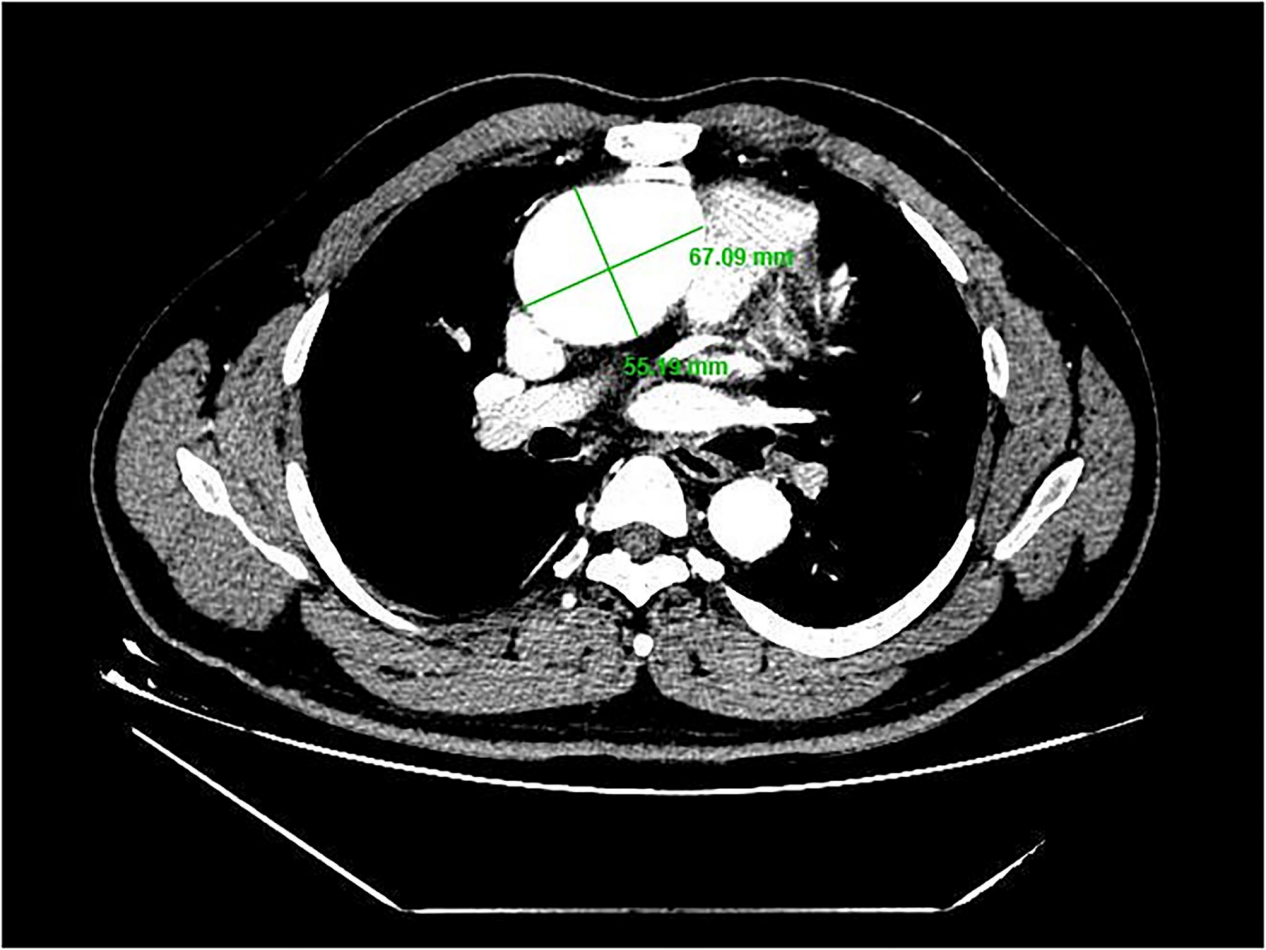
Diameter of aortic root 55.19 mm × 67.09 mm.

A 39-year-old male patient was admitted, complaining of persistent hemoptysis for 3 weeks. The patient was a typical TOF, not TOF of SD I type. Previously, he underwent radical surgery for tetralogy of Fallot, including relief of right ventricular outflow tract obstruction and repair of ventricular septal defect at 21. A diastolic murmur was found in the second left intercostal space of the sternum and other physical examinations were negative. Based on findings observed from cardiac echocardiography, it was diagnosed as dilatation for both sinotubular junction and ascending aorta (annulus, 3.17 cm; sinus, max. 5.82 cm; proximal ascending aorta, 4.14 cm) and aortic valve insufficiency complicated with severe AR (Figure 1A). The preoperative thoracic CT scan showed the diameter of the aortic root was 55.19 mm × 67.09 mm (Figure 2). Meanwhile, local obstruction of LVOT was also detected (Figure 1B), the gradients of LVOT obstruction was 23 mmHg. After clinical assessments, without any contradictions, this patient received the procedure including both David I plus repaired aortic valve procedure, combined with simultaneous reconstruction of LVOT.

**Figure 3:**
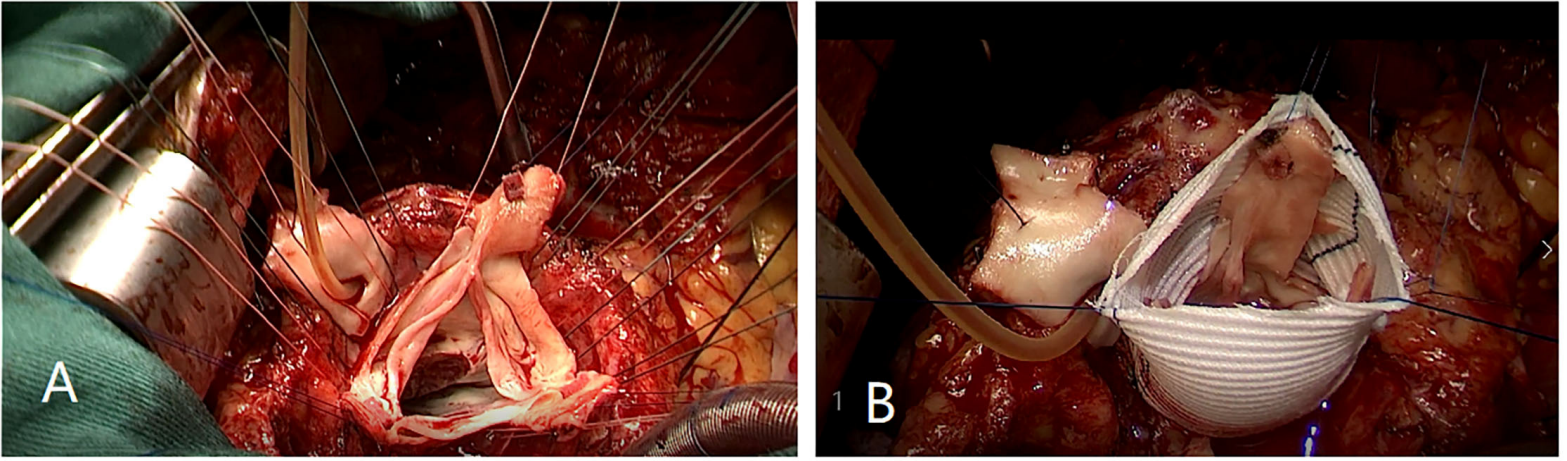
**(A)** Aortic root was cut 3 mm above annulus. **(B)** Each cusp of leaflets was fixed within the artificial graft by horizontal mattress suture during proximal anastomosis.

**Figure 4:**
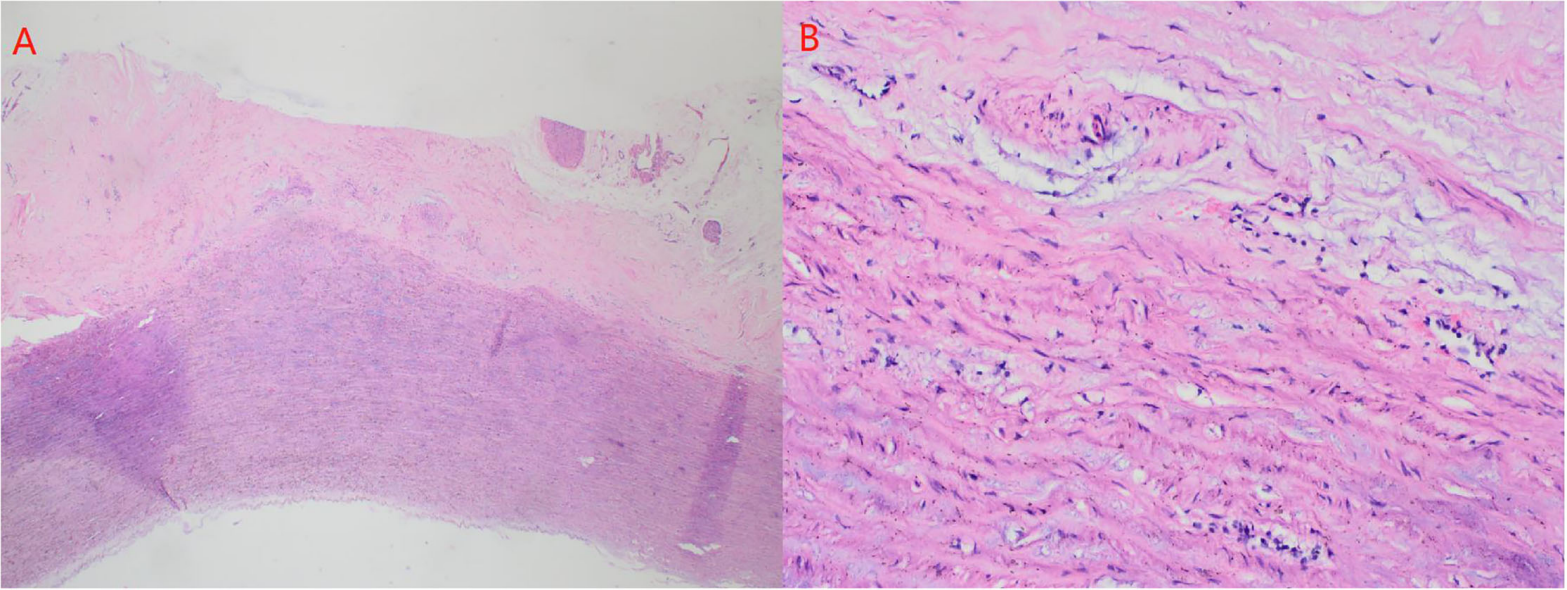
**(A)** atrophy and degeneration of smooth muscle in media of aortic wall (A × 50, hematoxylin, and eosin stain). **(B)** focal vitreous degeneration, myxoid degeneration (B × 200, hematoxylin, and eosin stain).

**Figure 5:**
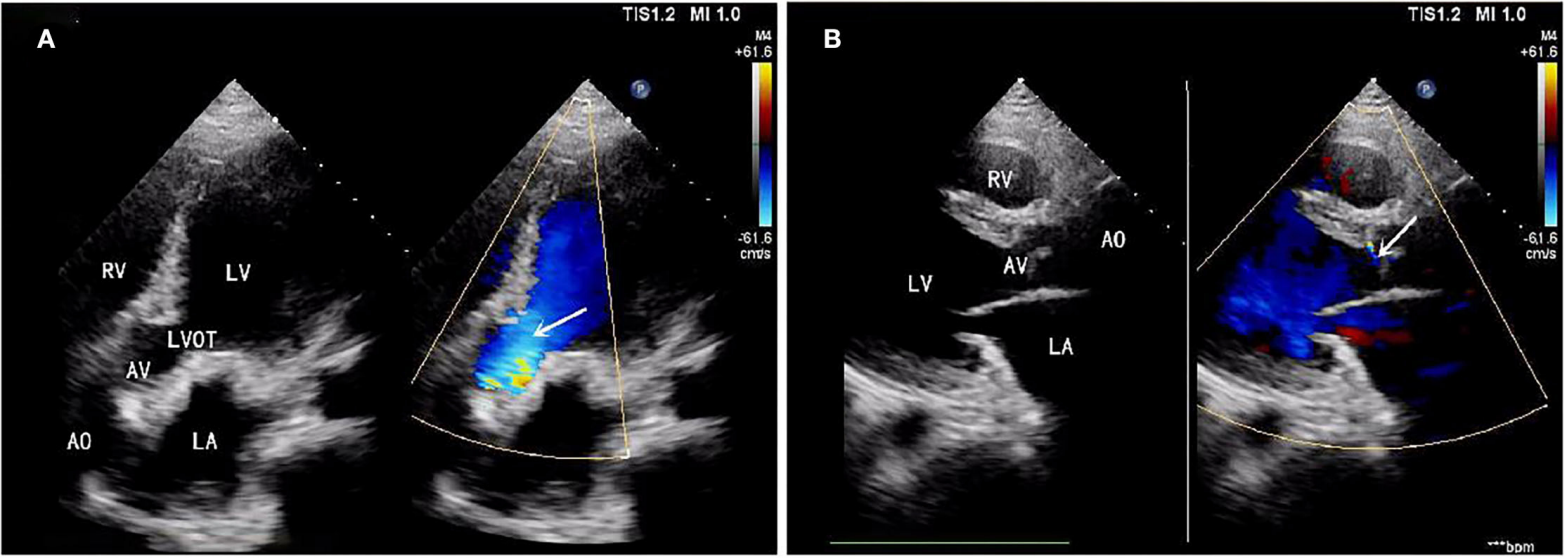
**(A)** Left ventricular outflow tract obstruction disappeared (Gradients of LVOT 7 mmHg). **(B)** Trivial aortic regurgitation.

Firstly, the separation of both femoral artery and vein was completed through the incision of the right groin. The median sternal incision was then taken and the separation for both superior and inferior vena cava was completed. Sequentially, the cardiopulmonary bypass (CPB) was established after stable catheterization into femoral artery and vein, as well as superior vena cava. The ascending aorta was clamped and Histidine-Tryptophan-Ketoglutarate solution (HTK) Cardioplegia was initiated at 34°C. Transection of the aorta at 1 cm above the sinotubular junction. Visually, dilatation of the aortic annulus and enlargement of the sinus were found. The aortic valve was not thickened, and leaflets were well-preserved. However, due to the dilatation of the aortic root, leaflets were mismatched with the aortic annulus, which will lead to misalignment of the aortic valves. After evaluating the function of the aortic valve, the thickened muscle bundles were seen in the left ventricular outflow tract *via* the aortic valve, and the thickened muscle bundles were removed with a fish abdominal blade. Then, the aortic root was separated for the measurement of the distance between the commissure of three leaflets and the annulus. Also, the distance between the midpoint and root of three leaflets was calculated at the same time. The aortic root, 3 mm above the annulus, was cut (Figure 3A) and a 32-mm artificial graft was selected. The height for commissure of leaflets was adjusted reasonably and each cusp of leaflets was fixed within the artificial graft by horizontal mattress suture during proximal anastomosis (Figure 3B). Anastomosis for both left and right coronary arteries to graft was completed by button technique. Lastly, distal anastomosis between the aorta and graft was performed. The ascending aorta was declamped after enough rewarming, the physiological heart rhythm was then activated automatically and smoothly. Then, no significant AR was found through intraoperative esophageal ultrasound. CPB was weaned when hemodynamics and vital signs were stable. Both regulatory chest closure and drainages for mediastinum of left and right chest cavities were performed without any active bleeding. The patient was transferred to the ICU ward for further medical supplements after surgery. The postoperative extubation was completed within the first 12 h in the ICU ward. It was detected that the size and shape of the graft were satisfactory and the function of the aortic valve was normal with mild regurgitation. Later, III° atrioventricular block was found by dynamic electrocardiogram, therefore, based on the consultant from a senior cardiologist and with the aim for improvement of rhythm, implantation of a permanent pacemaker was performed. Finally, the patient discharged after systematic postoperative clinical assessments were stable and qualified. Postoperative pathology showed atrophy and degeneration of smooth muscle in media of the aortic wall, focal vitreous degeneration, myxoid degeneration, and calcium salt deposition (Figure 4). The follow-up echocardiogram 2 months after the operation showed significant improvement in local obstruction of LVOT (Gradients of postoperative LVOT 7 mmHg), with trivial aortic regurgitation (Figure 5). During the follow-up of 1 year after surgery, the surgical effect is still positive and confirmed without any significant worse progression.

## Discussion

In a prospective trial, larger LV stroke volume has been found as an independent risk factor for delayed dilatation of aortic root due to increased shear stress (4). Based on the findings from 4D flow MRI, it has been suggested that among the patients after rTOF, elevated stiffness for both ascending and descending aorta is more significant and the wall shear stress (WSS) increases following physical growth. Although the diameter of the aorta remains in the normal range for a short time after the first repair of TOF, late dilatation of the aortic root and damage to the aortic wall are still inevitable (5). Moreover, up to the median of 7 years since the first repair, aortic stiffness is related to the incidence and severity of aortic root dilatation (6). Besides, François and colleagues, in their study to explore the histologic alterations of aortic root for infants after rTOF, have found in all selected samples of aorta, fibrosis, increased mucoid accumulation, and elastin fragmentation account for 45, 15, and 5%, respectively, and within 2 years after repairing, the regression of aortic diameters is significant (7). Chowdhury et al. (8) described that both substantial lamellar loss and intrinsic aortopathy were considered critical factors to the development of aortic abnormalities including degeneration, regression, and dilatation, and it was supposed that histologic alterations were correlated with the remodeling of ascending aorta. In this case, it had been 18 years for the patient in our report since initial TOF repairing, therefore, the structural abnormalities of the aortic root were progressive under the persistent negative influence of WSS. Meanwhile, the dysfunction and hypertrophy of LV were also presented due to elevated afterload and abnormal LV flow tract. Possibly, stiffness, as one explained the mechanism of aortic dilatation, reduces the flexible luminal expansion, and leads to elevated WSS, then, finally, induces turbulent flow within the aorta. Postoperative pathological results also showed atrophy and degeneration of aortic medial smooth muscle, focal vitreous degeneration, myxoid degeneration, and calcium salt deposition (Figure 4). In terms of organization and structure, this also confirms our assumption about the mechanism of progressive dilatation of aortic root after the operation of tetralogy of Fallot in this patient. We believe that the postoperative progressive dilatation of aortic root after rTOF is the leading cause for postoperative AR mainly associated with altered hemodynamics and inherent defects of the aortic wall. During the follow-up period, aortic dilatation has been found in most adult patients with rTOF, interestingly, however, it is concepted that as a part of normal physical process, not all aortic dilatation cause AR inevitably (9). This may be due to the dilatation of the aortic root did not involve the aortic annulus. It was worth mentioning that this patient performed CTA but not MRI preoperative. However, in a prospective cardiovascular MRI study, it has been recommended that as a comprehensive and accurate screening tool, with the aim of preventing TOF-associated aortic complications, cardiovascular MRI after rTOF is more necessarily needed to assess the diameter of the aortic root and detect residual of outflow tract, as well as structural data of right and left ventriculars (10).

Commonly, surgical intervention is the primary option when the diagnosis of aortic complications after rTOF is confirmed. Whereas the risks for both postoperative complications and early mortality are significantly higher, therefore, preoperative assessment should be emphasized (11). Currently, the recommended threshold of ascending aorta for surgical intervention is set as 55 mm (12). Bentall procedure is now the regular surgical skill for medical intervention of aortic complications with the advantages of improvement of LV function and decreased LV mass based on findings through long-term follow-up (13). Valve–sparing aortic replacement (VSRR) is an effective and safe surgical technique for pediatric patients with an aneurysms. Compared with the Bentall procedure and bioprosthetic valve conduit (BVC) replacement, the outcome after VSRR shows similar excellent survival and freedom from aortic re-intervention rates up to 10 years (14). Nevertheless, it is emphasized that the aortic valve can be spared instead of replaced mechanical or biological prosthesis, hence, both anticoagulation and degeneration of replaced prosthesis are avoided reasonably (15, 16). David I-V procedures are updated from the original VSRR. For this younger patient, David I plus repaired aortic valve procedure, combined with simultaneous reconstruction of LVOT, was selected after preoperative assessment and individual considerations, which could be an ideal and radical solution for aortic diseases with lower mortality and prevent adverse events after surgery, especially for anticoagulation-associated bleeding.

## Conclusion

Long-term follow-up of aortic root and valve is necessary for patients with rTOF. Any aortic complications, such as dilatation of aorta root or ascending aorta, and AR can be detected by imaging MRI. As an alternative skill for both Bentall and BVC, David's procedure is a flexible and critical component for surgical intervention to improve dilatation of aortic root and severe AR after rTOF, which maintains stable hemodynamics within the aorta and morphological characteristics of the aorta and without anticoagulation-associated serious adverse events.

## Data Availability Statement

The original contributions presented in the study are included in the article/Supplementary Materials, further inquiries can be directed to the corresponding author.

## Ethics Statement

Written informed consent was obtained from the individual(s) for the publication of any potentially identifiable images or data included in this article.

## Author Contributions

SZ and HL: collected relevant data and drafted the manuscript. XW and SH: taking pictures. CZ and HL: revised the manuscript. CZ and SZ: supervised the audit process. All authors read and approved the final manuscript.

## Conflict of Interest

The authors declare that the research was conducted in the absence of any commercial or financial relationships that could be construed as a potential conflict of interest.

## Publisher's Note

All claims expressed in this article are solely those of the authors and do not necessarily represent those of their affiliated organizations, or those of the publisher, the editors and the reviewers. Any product that may be evaluated in this article, or claim that may be made by its manufacturer, is not guaranteed or endorsed by the publisher.
