# Supplementary figures and images for: The Surgical Strategy for Progressive Dilatation of Aortic Root and Aortic Regurgitation After Repaired Tetralogy of Fallot: A Case Report
Source: Front Cardiovasc Med. 2022 May 3;9:840946. doi: 10.3389/fcvm.2022.840946 (PMC9110687; doi:10.3389/fcvm.2022.840946)

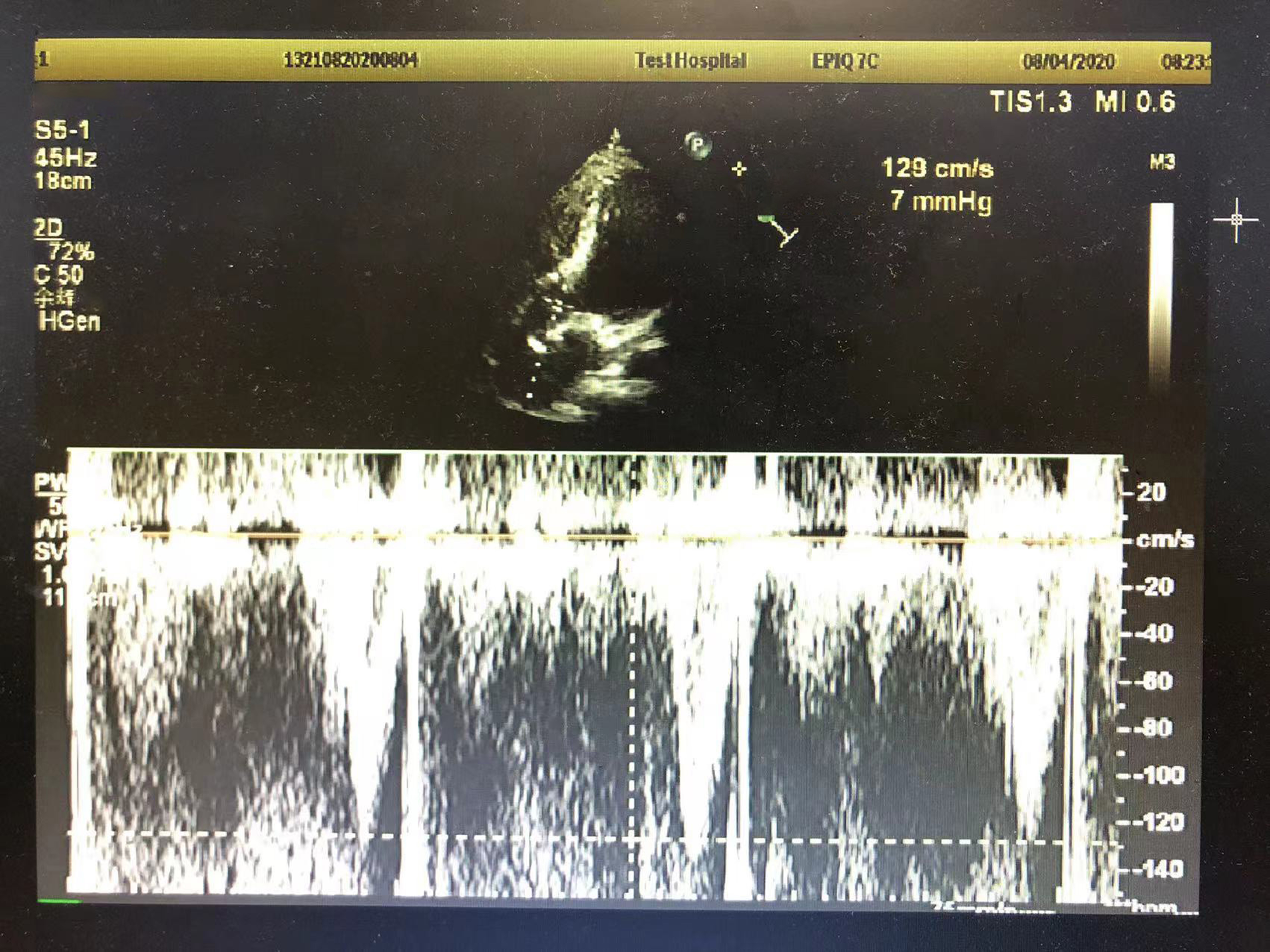

Supplement: Supplementary file 1 [file Image_1.JPEG]
